# Evaluation of magnetic stimulation as a non-invasive technique in treating different causes of erectile dysfunction: a prospective cohort study

**DOI:** 10.1186/s12610-025-00272-3

**Published:** 2025-06-20

**Authors:** Hasan El-Fakahany, Haythem Bassyouni, Sameh Fayek GamalEl Din, Mahmoud H. A. Montaser

**Affiliations:** 1 Department of Dermatology, STDs & Andrology, Faculty of Medicine, Minia University, Minia, Egypt; 2 Department of Andrology & STDs Kasr Alainy Faculty of Medicine, Cairo University, Al-Saray Street, El Manial, Cairo, 11956 Egypt

**Keywords:** Functional magnetic stimulator, IIEF-15, Arteriogenic ED, Veno-occlusive ED, Psychogenic ED, Stimulateur magnétique fonctionnel, IIEF-15, DE artériogène, DE veino-occlusive, Dysfonction érectile (DE) psychogène

## Abstract

**Background:**

Elaboration of alternative therapeutic modality is needed, which should be safer, less costly and with no side effects. Functional Magnetic Stimulator (FMS) is a technique that was approved by the US Food and Drug Administration in 1998. We aimed to evaluate the efficacy of FMS in treating different causes of erectile dysfunction (ED).

**Results:**

The mean baseline 15 items of the international index of erectile function (IIEF-15) scores of groups A (arteriogenic ED), B (veno-occlusive ED) and C (psychogenic ED) were 12.8 ± 2.6, 16.2 ± 3.3 and 27.5 ± 3.7, respectively. The mean post sessions IIEF-15 scores of groups A, B and C were 23.4 ± 4.1, 31.5 ± 3.5, 49.2 ± 3.5, respectively. The mean baseline domains of erectile function (EF) scores of groups A, B and C were 3.8 ± 2, 5.8 ± 3.5 and 11.5 ± 3.1, respectively. The mean post sessions domains of EF scores of groups A, B and C were 10.6 ± 3.1, 15 ± 3.6 and 24.8 ± 2.9, respectively. The mean baseline domains of orgasmic function scores of groups A, B and C were 3.3 ± 0.9, 3.9 ± 0.5 and 4.8 ± 0.4, respectively. The mean post sessions domains of orgasmic function scores of groups A, B and C were 4.8 ± 0.8 5.9 ± 1.0 and 8.6 ± 0.5, respectively. The mean baseline domains of sexual desire scores of groups A, B and C were 2.6 ± 0.6, 3.1 ± 0.7 and 4.8 ± 0.7, respectively. The mean post sessions domains of sexual desire scores of groups A, B and C were 3.6 ± 0.5, 4.4 ± 0.7 and 6.6 ± 0.6, respectively. The mean baseline domains of intercourse satisfaction scores of groups A, B and C were 1.9 ± 0.5, 2.4 ± 0.6 and 4.1 ± 0.6, respectively. The mean post sessions domains of intercourse satisfaction scores of groups A, B and C were 2.6 ± 0.5, 3.6 ± 0.5 and 5.5 ± 0.6, respectively.

The baseline domains of overall satisfaction scores of groups A, B and C were 1.1 ± 0.3, 1.1 ± 0.3 and 2.4 ± 0.7, respectively. The mean post sessions domains of overall satisfaction scores of groups A, B and C were 2 ± 0.7, 2.7 ± 0.7 and 3.8 ± 0.7, respectively.

**Conclusions:**

All patients showed significant improvement regarding domains of the IIEF-15 especially psychogenic cases following the sessions of FMS. Future studies should demonstrate the impact of these sessions on the response of poor responders to phosphodiesterase inhibitors and intracorporeal injections.

## Résumé

**Contexte:**

L’élaboration d’une modalité thérapeutique alternative est nécessaire, qui devrait être plus sûre, moins coûteuse et sans effets secondaires. Le stimulateur magnétique fonctionnel (SMF) est une technique qui a été approuvée par la Food and Drug Administration des États-Unis en 1998. Notre objectif était d’évaluer l’efficacité du FMS dans le traitement de différentes causes de dysfonction érectile (DE).

**Résultats:**

La moyenne de base de l’indice international de la fonction érectile (IIEF-15) des 15 éléments dans les groupes A (DE artériogène), B (DE veino-occlusif) et C (DE psychogène) était respectivement de 12,8±2,6, 16,2± 3,3 et 27,5±3,7. La moyenne des scores de l’IIEF-15 après les séances des groupes A, B et C était respectivement de 23,4±4,1, 31,5±3,5 et 49,2±3,5.

La moyenne des scores de base de la fonction érectile (FE) dans les groupes A, B et C était de 3,8± 2, 5,8±3,5 et 11,5±3,1 respectivement. La moyenne des scores de la FE après les séances était dans les groupes A, B et C respectivement de 10,6±3,1, 15±3,6 et 24,8±2,9.

La moyenne des scores de base des domaines de la fonction orgasmique était respectivement dans les groupes A, B et C de 3,3±0,9, 3,9±0,5 et 4,8±0,4. La moyenne des scores des domaines de la fonction orgasmique après les séances était respectivement dans les groupes A, B et C de 4,8±0,8, 5,9±1,0 et 8,6±0,5.

La moyenne des scores de base des domaines du désir sexuel des groupes A, B et C était de 2,6±0,6, 3,1±0,7 et 4,8±0,7, respectivement. La moyenne des scores des domaines du désir sexuel après les séances était respectivement dans les groupes A, B et C de 3,6±0,5, 4,4±0,7 et 6,6±0,6.

La moyenne des scores de base des domaines de la satisfaction sexuelle dans les groupes A, B et C était de 1,9±0,5, 2,4±0,6 et 4,1±0,6, respectivement. La moyenne des scores des domaines de la satisfaction sexuelle après les séances était respectivement dans les groupes A, B et C de 2,6±0,5, 3,6±0,5 et 5,5±0,6.

La moyenne des scores de base des domaines de référence de la satisfaction globale des groupes A, B et C était respectivement de 1,1±0,3, 1,1±0,3 et 2,4±0,7. La moyenne des scores des domaines de référence de la satisfaction globale après les séances des groupes A, B et C était respectivement de  2±0,7, 2,7±0,7 et 3,8±0,7.

**Conclusions:**

Tous les patients ont montré une amélioration significative en ce qui concerne les domaines de l’IIEF-15, en particulier les cas psychogènes, après les séances de FMS. De futures études devraient démontrer l’impact de ces séances sur la réponse des hommes peu répondeurs aux inhibiteurs de la phosphodiestérase et aux injections intracorporelles.

## Introduction

Erectile dysfunction (ED) is the persistent inability to achieve or maintain an erection that is appropriate for satisfactory sexual relationship. By 2025, the number of ED cases is expected to reach 322 million worldwide [1]. Veno-occlusive dysfunction (VOD) might be attributed to aging or changes to the tunica albuginea due to injury, cavernosal smooth muscle dysfunction due to anatomical alterations, excessive adrenergic input or from priapism episodes and subsequent repair [2, 3]. Although the underlying reasons of VOD are not yet fully elucidated, several situations including aging, diabetes, and androgen deprivation therapy appear to be potential risk factors [4]. Corona et al. (2016) stated that high TG levels are associated with arteriogenic ED and with an increased risk of forthcoming cardiovascular events due to impairment of nitric oxide mediated erectile function [5]. Moreover, it should be noted that diabetes-associated microangiopathy was clearly associated with a higher risk of major adverse cardiovascular events, especially in young and less complicated patients [6, 7]. Thus, it could be speculated that hypertriglyceridemia also might play a role in the ED-associated structural changes of penile vessels [5, 8]. Meanwhile, patients with primary psychogenic ED experienced various degrees of psychological distress, because of performance anxiety due to the inability to obtain and maintain an erection adequate for satisfactory sexual intercourse [9–11]. Henceforth, the European association of urology (EAU) Guidelines on Sexual and Reproductive Health reclassified ED into primary organic and primary psychogenic [12].

In view of medical therapy, phosphodiesterase type 5 inhibitors (PDE5Is) had revolutionized treatment of ED and were considered the first therapeutic option for ED [13]. However, this type of treatment was not accepted by all patients [4]. Penile prosthesis is the surgical option which maintains its significance as a cure for ED. There are convincing long-term results with a high degree of patient’s and his partner’s satisfaction, high patient acceptance, and good functional durability of the penile prothesis. However, some patients do not prefer this option because of its high cost [14]. Elaboration of alternative therapeutic modality is needed, which should be safer, less costly and with no side effects. Functional Magnetic Stimulator (FMS) is a technique that was approved by the US Food and Drug Administration in 1998 [15]. FMS was originally used for treating women with mixed urinary incontinence [16]. The mechanism of action was postulated via magnetic stimulation of the peripheral nerves and muscles of the pelvis [17–19]. Notably, Shafik et al. 2000 [20] was one of the first to demonstrate the favourable effect of MS on the cavernous tissue to induce penile rigidity in humans. Furthermore, a recent study evaluated the effect of MS on 20 patients with ED and showed a good response of the ED patients without side effects [21]. The present study aimed to evaluate the efficacy of FMS in treating different causes of ED.

## Patients and methods

The study was conducted on 180 adult male patients complaining of ED attending andrology outpatient clinic of Elmania University Hospital from December 2018 to December 2019.

Approval of the committee for postgraduate studies and research of Elmania University (approval number: 150:/2019) was obtained that conformed to Helsinki declaration 2013 [22]. Patients were equally divided into 3 groups, group A included subjects with arteriogenic ED, whereas group B included subjects with veno-occlusive ED and group C included subjects with psychogenic ED.

### Inclusion criteria of the patients

Any patient with ED due to arteriogenic ED or veno-occlusive dysfunction ED and was non responder to PDE-5Is and intracorporeal injection (ICI) was included. Also, subjects with psychogenic ED were included.

### Exclusion criteria of the patients

Patients with ED due to chronic illnesses including ischemic heart disease, renal failure, or patients with neurogenic ED were excluded. Also, patients who did not stop PDE-5Is and ICI during the FMS sessions were excluded.

All participants were subjected to the following:

Medical and surgical histories were obtained. General and local examinations were done. FMS (TESLA Stym, Iskra Medical, Ljubljana, Slovenia) was used. The procedure was done in the supine position without any anesthesia. Every patient underwent 40 sessions, twice weekly over 5 months and every subject was assessed for erectile function by the 15 questions of the International Index of Erectile Function (IIEF-15) before and after the procedure that was self-administered in a structured interview [23].

**Fig. 1:**
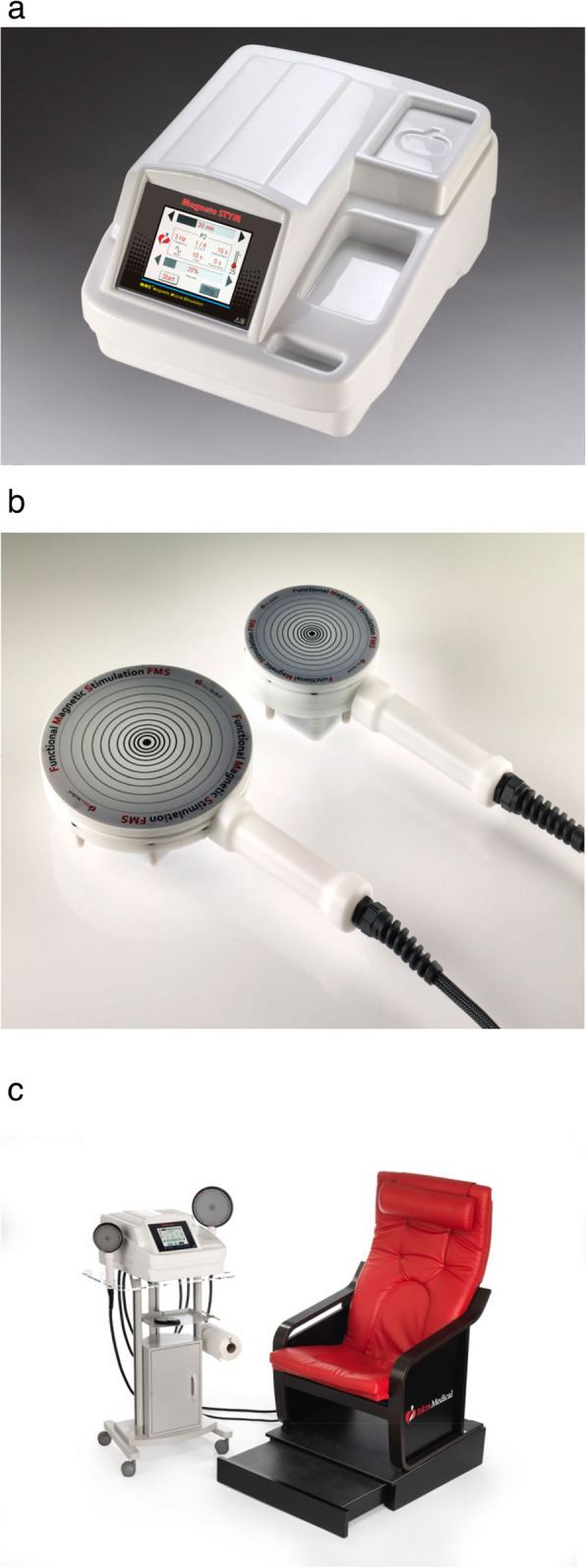
**a** shows the external unit of FMS. **b** shows mobile FMS applicator or FMS hand peace. **c** shows FMS Chair

All participants underwent penile duplex to confirm the diagnosis of their condition using 0.25 cc prostaglandin-E 1 (PGE1, prostin VR Pfizer) [24]. The penile duplex readings for detecting different types of ED were set according to the data revealed by Aversa and Sarteschi (2007) and Ioakeimidis et al., (2011) [25, 26]. The degree of erection of the participants was evaluated by the erection hardness scale [27]. FMS is composed of 3 units. The main external unit (Fig. 1a) by which the magnetic field can be controlled by adjusting the frequency and amplitude of stimulation. The amplitude determines the volume of the field, and whether it is strong enough to induce a nerve impulse. Mobile FMS applicator or FMS hand peace (Fig. 1b) consists of multiple magnetic coils in a hand peace which imports magnetic pulses from the main device. The FMS applicator is placed on the dorsal aspect of the penis. Thus, it may stimulate not only the cavernous nerve but also the deep dorsal nerve of the penis. FMS chair (Fig. 1c) is a medical device which uses electromagnetic pulsing technology for pelvic floor muscle stimulation. It generates a homogeneous magnetic field via a magnetic coil embedded beneath the surface of the seat. Finally, drop out cases were not noticed in the study as this machine was non-invasive and all patients felt good response following these sessions. Additionally, all participants did not need to repeat penile duplex after improvement in their erectile function evidenced by the IIEF-15 evaluation at the end of the study.

#### Step 1 Hand peace session

The stimulation was provided by the mobile FMS applicator (Fig. 1b), which was placed on the dorsal aspect of the penis and controlled by the external unit (Fig. 1a). FMS was performed using a stimulation of 60% intensity, 10 Hz frequency and 10 s on and 10 s off for 10 min duration followed by 60% intensity, 25 Hz frequency and 10 s on and 10 s off for 10 min. These parameters provided penile stimulation without frequent over-heating the magnetic coil.

#### Step 2 Chair session

Patients sat on the FMS chair (Fig. 1c), an electromagnetic generator in the seat, controlled by the external unit, provided stimulation. The effect was greatest in the center of the field and therefore the perineum was centered in the middle of the seat. FMS was performed using a stimulation of 60% intensity, 10 Hz frequency and 10 s on and 10 s off for 10 min duration followed by 60% intensity, 25 Hz frequency and 10 s on and 10 s off for 10 min.

### Statistical analysis

The statistical analysis was conducted using IBM SPSS version 27.0 (IBM Corp., Armonk, NY, USA). Quantitative data were expressed as means and standard deviations (SD). Normality of distribution was assessed using the Shapiro–Wilk test. Since the data were normally distributed, parametric tests were applied. Paired sample t-test was used to compare pre-treatment and post-treatment scores.

### Sample size determination

The minimum sample size was calculated using the G*Power software 3.1.9.7; (Heinrich-Heine-Universität Düsseldorf, Düsseldorf, Germany). Based on a priori analysis for t test (difference between two dependent means; matched pairs) with an effect size of 0.4, significance level of 0.05, and power of 0.80, the projected required sample size was calculated to be 52. Considering dropout rate of 15% additional 8 patients were included in the analysis, providing 60 patients for each matched pair.

## Results

The mean age of the patients was 42.2 years ± 16.5. The mean baseline 15 items of the international index of erectile function (IIEF-15) scores of groups A (arteriogenic ED), B (veno-occlusive ED) and C (psychogenic ED) were 12.8 ± 2.6, 16.2 ± 3.3 and 27.5 ± 3.7, respectively (Table 1). The mean post sessions IIEF-15 scores of groups A, B and C were 23.4 ± 4.1, 31.5 ± 3.5, 49.2 ± 3.5, respectively (Table 1). The mean baseline domains of erectile function (EF) scores of groups A, B and C were 3.8 ± 2, 5.8 ± 3.5 and 11.5 ± 3.1, respectively (Table 2). The mean post sessions domains of EF scores of groups A, B and C were 10.6 ± 3.1, 15 ± 3.6 and 24.8 ± 2.9, respectively (Table 2). The mean baseline domain orgasmic function scores of groups A, B and C were 3.3 ± 0.9, 3.9 ± 0.5 and 4.8 ± 0.4, respectively (Table 2). The mean post sessions domain orgasmic function scores of groups A, B and C were 4.8 ± 0.8 5.9 ± 1.0 and 8.6 ± 0.5, respectively (Table 2). The mean baseline domain sexual desire scores of groups A, B and C were 2.6 ± 0.6, 3.1 ± 0.7 and 4.8 ± 0.7, respectively (Table 2).

**Table 1:** Shows the mean baseline and post sessions IIEF-15^a^ scores

	Group A (diabetic ED)	Group B (venogenic ED)	Group C (psychogenic ED)
Baseline			
Range (score)	8–18	10–22	20–35
Mean	12.8	16.2	27.5
SD^b^	± 2.6	± 3.3	± 3.7
Post sessions			
Range (score)	18–31	24–39	42–54
Mean	23.4	31.5	49.2
SD^b^	± 4.1	± 3.5	± 3.5
*P* value	*p* < 0.001	*p* < 0.001	*p* < 0.001

N.B: ^a^*IIEF-15* The 15-questions of the International Index of Erectile Function, ^b^*SD* standard deviation, *p* value was calculated using a paired sample t-test

**Table 2:** Shows mean scores of baseline and post sessions of all domains of 15-IIEF^a^

	Baseline			Post sessions			*P* value
	Range	Mean	SD^c^	Range	Mean	SD^c^	
Group A							
EF^b^	1–8	3.8	± 2	7–16	10.6	3.1 ±	< 0.001
Orgasmic function	2–4	3.3	± 0.9	4–6	4.8	± 0.8	
Sexual desire	2–4	2.6	± 0.6	3–4	3.6	± 0.5	
Intercourse satisfaction	1–3	1.9	± 0.5	2–3	2.6	± 0.5	
Overall satisfaction	1–2	1.3	0.5	1–3	2	0.7	
Group B							
EF^b^	2–10	5.8	± 3.2	9–20	15	± 3.6	< 0.001
Orgasmic function	3–5	3.9	± 0.5	4–7	5.9	± 1	
Sexual desire	2–4	3.1	± 0.7	3–5	4.4	± 0.7	
Intercourse satisfaction	2–4	2.4	± 0.6	3–4	3.6	± 0.5	
Overall satisfaction	1–2	1.1	0.3	1–4	2.7	0.7	
Group C							
EF^b^	8–16	11.5	± 3.1	19–28	24.8	± 2.9	< 0.001
Orgasmic function	4–5	4.8	± 0.4	8–9	8.6	± 0.5	
Sexual desire	4–6	4.8	± 0.7	6–8	6.6	± 0.6	
Intercourse satisfaction	3–5	4.1	± 0.6	5–7	5.5	± 0.6	
Overall satisfaction	1–4	2.4	0.7	3–6	3.8	0.7	

N.B: ^a^*IIEF-15* The 15-questions of the International Index of Erectile Function, ^b^*EF* erectile function, ^c^*SD* standard deviation, *p* value was calculated using a paired sample t-test

The mean post sessions domain sexual desire scores of groups A, B and C were 3.6 ± 0.5, 4.4 ± 0.7 and 6.6 ± 0.6, respectively (Table 2). The mean baseline domain intercourse satisfaction scores of groups A, B and C were 1.9 ± 0.5, 2.4 ± 0.6 and 4.1 ± 0.6, respectively (Table 2). The mean post sessions domain intercourse satisfaction scores of groups A, B and C were 2.6 ± 0.5, 3.6 ± 0.5 and 5.5 ± 0.6, respectively (Table 2). The baseline domain overall satisfaction scores of groups A, B and C were 1.1 ± 0.3, 1.1 ± 0.3 and 2.4 ± 0.7, respectively (Table 2). The mean post sessions overall satisfaction scores of groups A, B and C were 2 ± 0.7, 2.7 ± 0.7 and 3.8 ± 0.7, respectively (Table 2). The baseline penile duplex readings were recorded and reported in Table 3.

**Table 3:** Shows the baseline penile duplex readings of the participants

	Group A Arteriogenic	Group B Venogenic	Group C Psychogenic
PSV^a^ (cm/s)			
Range	15.2–24.6	25.4–39.6	25.4–39.6
Mean	19.6	32.1	32.1
± SD	± 3	± 4.5	± 4.5
EDV^b^ (cm/s)			
Range	1–4.9	6.1–14.9	0.1–4.9
Mean	2.9	10.4	2.5
± SD	± 1.2	± 2.6	± 1.4
RI^c^			
Range	0.7–1	0.5–0.8	0.8–1
Mean	0.8	0.7	0.9
± SD	0.1	0.1	0.04

N.B: ^a^*PSV* peak systolic velocity, ^b^*EDV* end diastolic velocity, ^c^*RI* resistive index

## Discussion

Our study demonstrated the favorable impact of FMS on the erectile function of the participants especially those with psychogenic ED due to utilizing parameters that provided adequate penile stimulation without frequent over-heating of the magnetic coil. In a similar trend, Barker et al. 1987 [28] reported that magnetic field near neuromuscular tissue induced electric field that can stimulate muscular tissue. FMS worked by creating a magnetic field that, according to Faraday's law, created an electric field that appeared to activate the cavernous nerve. It could stimulate both cavernous nerves which likely caused smooth muscles that surround helical arterioles and lacunar spaces of the penis to relax. Additionally, FMS applicator could stimulate deep dorsal nerve of the penis efferent of the dorsal nerve that controlled the blood vessels in the skin of the penis or modulated the sensitivity of the afferent receptors [29, 30].

Further, stimulation of the somatic nerves with one-off mode causes contraction and relaxation of ischiocavernosus and bulbospongiosus muscle resulting in strengthening of these muscles and increasing their vascularity [30]. It should be mentioned that Shafik et al. 2000 [20] was one of the first to highlight the positive effect of MS on the cavernous tissue to induce penile rigidity in humans. Nevertheless, our technique differed from Shafik et al. 2000 [20] who placed the magnetic coil on the dorsal aspect of the penis in the premises of the symphysis pubis utilizing a stimulation of 40% intensity, 20 Hz frequency, 50 s on and 50 s off for 10 min duration. Furthermore, a recent study evaluated the effect of MS on 20 patients with ED and showed that it was an effective therapy for ED without side effects [21]. The aforementioned study utilized a different technique that was scheduled as follows: sessions 1 to 4 followed the Hypotonus/Weakness 1 protocol whereas sessions 5 to 8 followed the Hypotonus/Weakness 2 protocol [21]. The Hypotonus/Weakness 1 protocol conducted around 30 min warm-up and muscle activation phase, then a muscle work phase was conducted that based on restoring volume and muscle tone (20-30Hz) in a trapezoidal shape [21]. These abovementioned steps were followed in the Hypotonus/Weakness 2 protocol, but a higher frequency (40-50Hz) was utilized in the muscle work phase as it aimed at increasing volume, and a muscle strength phase [21]. Endothelial dysfunction is a central player of ED [31] as well as a muscular involvement [21]. It should be mentioned that the marvelous response observed in our patients might be attributed to the favorable effect of FMS on different skeletal muscles [21].

In a similar trend, Leone et al. (2021) demonstrated hypertrophy in terms of abdominal muscle tissue thickness of their studied patients following a device that utilized FMS technology [32]. Remarkably, smooth muscle tissue represented around 45% of the cavernous volume, with collagen represented the major portion of the non-muscle component with the smooth muscle of the penis playing the essential role of the hemodynamic processes that underlie an erection [21]. Thus, magnetic stimulation might be essential in treating ED, ameliorating fibromuscular pathological changes within the corpus cavernosum [21]. Karacan et al. (1983) had revealed that bursts in the perineal muscles'EMG activity during nocturnal penile tumescence coincided with peaks in blood flow recordings [33]. Consistently, a review of literature conducted by Van Kampen et al. (2003) showed that perineal rehabilitation could be an effective treatment for ED [34]. It could be rephrased that the findings of Galimberti et al. (2024) [21] endorsed the hypothesis that pelvic floor rehabilitation played a part in improving ED that could be seen in line with those of Rival and Clapeau (2017) [35]. In a similar trend, a study had postulated utilizing perineal rehabilitation as a first-line treatment for ED [36]. FMS could decrease the production of the pro-inflammatory cytokine TNF-alpha and increase the production of the anti-inflammatory cytokine IL-10 by shifting astrocytic phenotypes (A1-A2) in animal models [21]. Furthermore, FMS also increased the release of angiogenesis-related factors TGFb and VEGF in A2 astrocytes, which could support angiogenesis [21]. A previous animal study demonstrated up-regulation of angiogenesis-related genes (VEGFA and BAI1) following FMS [37]. In the same context, Lee and colleagues (2022) demonstrated changes in the angiogenic pathways following magnetic stimulation, on the affected hemisphere in an animal model with a stroke [38]. Factually, it should be mentioned that MS significantly increased endothelial nitric oxide synthase phosphorylation, which stimulated angiogenesis [38]. In view of the abovementioned facts, it could be postulated that FMS had positive muscular effects as well as probable NO-dependent benefits in angiogenesis, making it a potential noninvasive therapeutic modality for psychogenic patients as well as patients with vasculogenic ED and poor response to PDE5I [21] or ICI as in our study. Additionally, a recent animal study had shown that trans-pelvic MS was a novel and non-invasive maneuver to enhance penile micro-vascular perfusion following cavernosal nerve injury [39]. Thus, it could be used in treating ED and penile rehabilitation following cancer treatment, probably through enhancing penile hemodynamics that might assist preventing penile hypoxia and fibrosis [39]. Once again, our study demonstrated a favorable impact of the FMS on all participants, especially those with psychogenic ED. Thus, FMS presented a non-invasive technique that enabled patients with psychogenic ED to restore their potency with the avoidance of offering invasive treatments such as ICI for a limited period or oral PDE5Is that might not be preferred by such patients. Similarly, Salonia et al. (2021) [12] and Pozzi et al. (2022) [40] stressed the necessity of accurate psychosexual counselling as the corner stone of managing psychogenic ED cases as well as a collaboration between a physician and a psychologist who should be experts in sexual medicine with the avoidance of offering invasive treatments.

### Limits of study

Admittedly, the short follow up period should be considered the main limitation and the inability to re-evaluate the patients’ response to PDEIs-5 and ICI following these sessions. Finally, we did not use the validated Arabic version of the international index of erectile function [41] as the construct validity of the IIEF-15 items version of the international index of erectile function [23] was good together with the ability to detect changes before and after treatment.

## Conclusion

All patients showed significant improvement regarding domains of the 15-IIEF especially psychogenic cases following the sessions of FMS. Thus, FMS may be an effective noninvasive therapeutic modality that can be utilized in treating different causes of ED. Future studies should demonstrate the impact of these sessions on the response of poor responders to PDE5Is and ICI as well as rehabilitating the sexual function of patients who underwent radical prostatectomy.

## Data Availability

No datasets were generated or analysed during the current study.

## Abbreviations

ED: Erectile dysfunction
EDV: End diastolic velocity
EF: Erectile function
FMS: Functional Magnetic Stimulator
ICI: Intracorporeal injection
IIEF-15: The 15 questions of the International Index of Erectile Function
PDE5Is: Phosphodiesterase type 5 inhibitors
PSV: Peak systolic velocity
RI: Resistive index
VOD: Veno-occlusive dysfunction
